# Fabrication of Recycled Polycarbonate Fibre for Thermal Signature Reduction in Camouflage Textiles

**DOI:** 10.3390/polym14101972

**Published:** 2022-05-12

**Authors:** Asril Soekoco, Ateeq Ur Rehman, Ajisetia Fauzi, Hamdi Tasya, Purnama Diandra, Islami Tasa, Nugraha, Brian Yuliarto

**Affiliations:** 1 Department of Textile Engineering, Politeknik STTT Bandung, Kota Bandung 40272, Indonesia; fauziaji98@gmail.com (A.F.); tasya.wirdati27@gmail.com (H.T.); diandra190896@gmail.com (P.D.); tasaislami7@gmail.com (I.T.); 2 Department of Engineering Physics, Faculty of Industrial Technology, Institut Teknologi Bandung, Kota Bandung 40132, Indonesia; nugraha@tf.itb.ac.id; 3 School of Engineering, College of Science & Engineering, The University of Edinburgh, Edinburgh, EH8 9YL, UK; ateeq-ur-rehman@sms.ed.ac.uk

**Keywords:** polycarbonate waste, recycled fibre, thermal signature, camouflage textiles

## Abstract

Thermal signature reduction in camouflage textiles is a vital requirement to protect soldiers from detection by thermal imaging equipment in low-light conditions. Thermal signature reduction can be achieved by decreasing the surface temperature of the subject by using a low thermally conductive material, such as polycarbonate, which contains bisphenol A. Polycarbonate is a hard type of plastic that generally ends up in dumps and landfills. Accordingly, there is a large amount of polycarbonate waste that needs to be managed to reduce its drawbacks to the environment. Polycarbonate waste has great potential to be used as a material for recycled fibre by the melt spinning method. In this research, polycarbonate roofing-sheet waste was extruded using a 2 mm diameter of spinnerette and a 14 mm barrel diameter in a 265 °C temperature process by using a lab-scale melt spinning machine at various plunger and take-up speeds. The fibres were then inserted into 1 × 1 rib-stitch knitted fabric made by Nm 15 polyacrylic commercial yarns, which were manufactured by a flat knitting machine. The results showed that applying recycled polycarbonate fibre as a fibre insertion in polyacrylic knitted fabric reduced the emitted infrared and thermal signature of the fabric.

## 1. Introduction

Heat migrates from hotter areas to cooler areas through thermal convection, thermal conduction, and thermal radiation [1]. This phenomenon is applied in every aspect of our daily lives, from the most simple parts of our routine to the most sophisticated military applications. The combination of heat migration from an object to the environment can create a thermal signature, measured by thermal imaging techniques [2]. Thermal signature is affected by the temperature of the object and background, and an effective way to reduce it is by reducing the emissivity [3,4]. The temperature difference (ΔT) between the object and background affects the thermal region in thermal imaging [5,6]. Thermal imaging is proven to produce an advanced spectral range that can be seen by a human and shows an obvious contrast between the environment and objects of high-temperature variance [7,8,9,10,11]. Currently, thermal imaging techniques are used incommercial activities, industrial processes and by the military. The military is one area that develops camouflage textiles to protect a soldier from detection by various equipment in many operations, including surveillance purposes at night or in low-light conditions [4]. Generally, there are several polymers used in camouflage textile materials because of their capability to minimize infrared visual detection by reducing the thermal signature of the soldier, one of them is polycarbonate.

Polycarbonate polymer was developed in 1953; it is tough, rigid, and has a relatively high melting temperature, from 225 °C to 250 °C, with a glass transition temperature of 145 °C. It also has flame retardant property, with an oxygen index of 26, in addition to thermal and electrical insulation [12]. Polycarbonate is made from a condensation reaction of bisphenol A with phosphorus (phosgene) in alkaline media [13]. The presence of bisphenol A makes polycarbonate capable of absorbing infrared, which can be utilized for military textile applications [13,14]. Due to the optical and mechanical properties of polycarbonate, Fujitsu developed and introduced polycarbonate optical fibre in 1986 [15]. Polycarbonate fibre was developing rapidly, particularly for high-temperature resistant polymer sensor applications, since it has a high glass transition temperature and high flexibility for bending. Since that time, polycarbonate fibre also started to develop rapidly in various applications such as high-temperature-resistant polycarbonate fibre, electrical conductive polycarbonate fibre (by adding multiwalled carbon nanotubes), and strain sensing polycarbonate fibre [15,16,17]. However, all of the existing polycarbonate fibres are developed from virgin polycarbonate material instead of waste polycarbonate material. 

The global polycarbonate market significantly increased from 2000 to 2010, and it is forecasted to increase to 5.5 million tons by 2024, due to the high demand in the electrical, electronics, and automotive industries [13,18,19]. Polycarbonate is a hard type of plastic that is not easily recycled and polycarbonate waste generally ends up in dumps and landfills [20]. Accordingly, there is a huge amount of polycarbonate waste available which needs to be managed to reduce its drawbacks to the environment. Therefore, the fabrication of polycarbonate sheet waste for thermal signature reduction application is quite promising, particularly for incorporating microstructures into apparel for visible and infrared camouflage used in the military [21].

## 2. Materials and Methods

**Figure 1:**
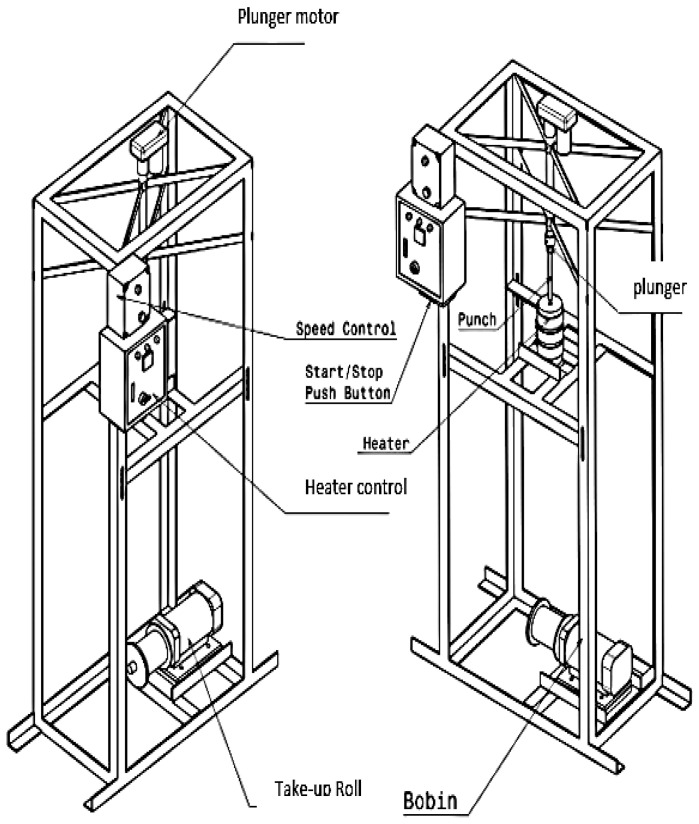
Schematic diagram of lab-scale melt spinning machine.

All of the polycarbonate fibres in this research were produced from multiwall polycarbonate roofing-sheet waste (grey colour, 5 mm thickness, from Twinlite, PT Impack Pratama, Jakarta, Indonesia). To identify and confirm the material, material characterization was conducted using an FTIR-8400 (Shimadzu, Kyoto, Japan). To produce recycled polycarbonate fibres, polycarbonate roofing-sheet waste was cleaned before the grinding process. The ground polycarbonate chips were then extruded using a 2 mm diameter of spinnerette and a 14 mm barrel diameter in a 265 °C temperature process, using a lab-scale melt spinning machine as can be seen in Figure 1. Variations in two processing parameters were applied to produce several polycarbonate fibre properties, plunger speed (0.10, 0.15 and 0.18 cm/s) and take-up speed (6.2, 6.9 and 9.2 m/min). The recycled polycarbonate fibres that were produced were then inserted into 1 × 1 rib-stitch knitted fabric made by Nm 15 polyacrylic commercial yarns, which were manufactured by a flat knitting machine with a fabric construction of 15 courses per inch and 18 wales per inch. 

The fibre diameter measurement was performed using an Olympus CX-22 binocular microscope (Tokyo, Japan) assisted camera adapter, the fibre tensile strength and elongation were measured using an Instron tensile testing machine according to ASTM D 3822-07, the fabric area density was measured according to SNI-ISO 3801:2010, and the fabric bursting test was measured according to SNI 0561:2008. To demonstrate the thermal signature reduction in fabrics, we first covered the heat source (40 °C, 68 °C and 100 °C) with the various polycarbonate–polyacrylic fabrics, the fabric surface temperature and the thermal signature of each fabric was then captured using a Flir One thermal imaging camera (Teledyne FLIR LLC, Wilsonville, OR, USA), which can measure temperatures from −20 °C to 200 °C (2% instrumental error). To achieve a high accuracy result, the ambient temperature was maintained between 30 °C and 45 °C, and the measurement distance was 0.8 m [22,23]. The surface temperature reduction in each fabric was calculated to discover the emissivity decrement and confirmed the thermal signature reduction effect.

## 3. Results and Discussion

### 3.1. Fabrication of Polyacrylic–Polycarbonate Fabric

**Figure 2:**
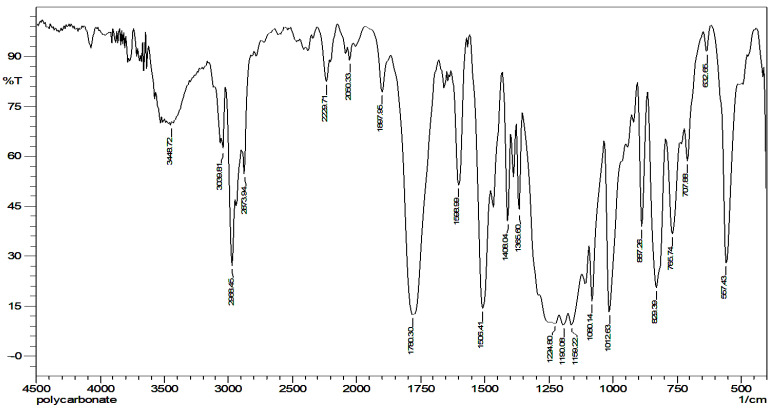
IR absorption spectrum of multiwall roofing-sheet waste.

The FTIR spectrum of polycarbonate sheet waste is shown in Figure 2. Polycarbonate has principal peaks around 1015 cm^−1^ caused by symmetric O–C–O carbonate group deformations, CH3-vibrations around 1081 cm^−1^, C=C-vibrations at 1506 cm^−1^, C=O carbonate group deformations near 1775 cm^−1^ and peaks around 3.000 cm^−1^ caused by C–H aromatic ring deformations. The result of the characterization confirmed that the polymer of this material was polycarbonate.

**Figure 3:**
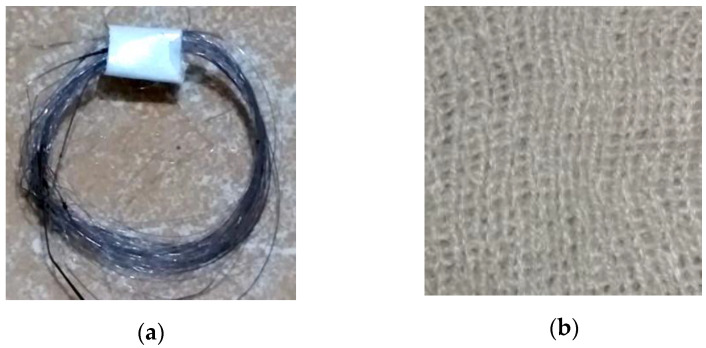

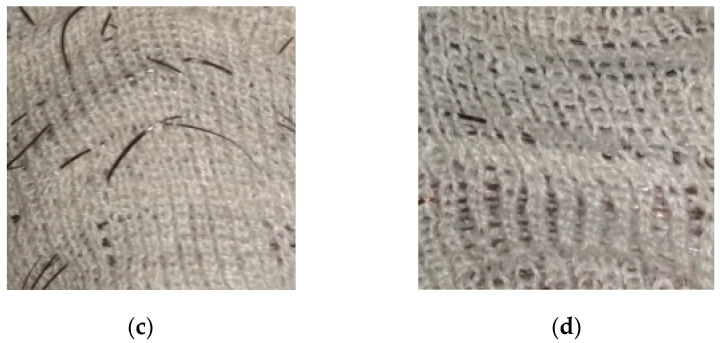
(**a**) Recyled polycarbonate fibre; (**b**) 0% Polycarbonate–100% Polyacrylic fabric; (**c**) 50% Polycarbonate–50% Polyacrylic; (**d**) 66.7% Polycarbonate–33.3% Polyacrylic.

Figure 3b–d show the several variations of fabric produced in this research, all of these variations were used in a 1 × 1 rib-stitch knit due to its structural dimension stability and extensive shrinkage in width [24]. To demonstrate the thermal signature reduction ability of the recycled polycarbonate fibre, we prepared three variations of polycarbonate composition in knitted fabric by inserting recycled polycarbonate fibres in polyacrylic knitted fabric. We prepared fabrics containing 0% polycarbonate–100% polyacrylic fabric (without polycarbonate fibre insertion); 50% polycarbonate–50% polyacrylic (with one strand of polycarbonate fibre insertion) and 66.7% polycarbonate–33.3% polyacrylic (with two strands of polycarbonate fibre insertion). This variation was the best option considering the structure of the 1 × 1 rib-stitch fabric construction and the fluency of the knitting process in knitted fabric fabrication, according to a preliminary research result. The amount of recycled polycarbonate fibre insertion influenced the fabrication process, excessive recycled polycarbonate fibre insertion caused a jam of the yarn feeding, whereas inadequate recycled polycarbonate fibre insertion may cause loose and stray fibre in the fabric due to improper yarn tension.

### 3.2. Fibre Diameter

In the second part of the study, we examined the correlation of plunger speed and take-up speed with the recycled polycarbonate fibre diameter. Considering the available machine settings, we used five variations of processing parameters; the highest plunger speed was 0.18 m/min and the lowest plunger speed was 0.10 m/min, whereas the highest take-up speed was 9.2 cm/min and the lowest plunger speed was 6.9 cm/min. We determined that a high take-up speed resulted in a finer fibre diameter than a low take-up speed. Meanwhile, a low plunger speed produced a coarser fibre diameter than a fast plunger speed. This occurred because of the strain rate along the spin line [25]. Higher take-up speed leads to a higher strain rate, but otherwise higher plunger speed leads to a lower strain rate.

**Figure 4:**
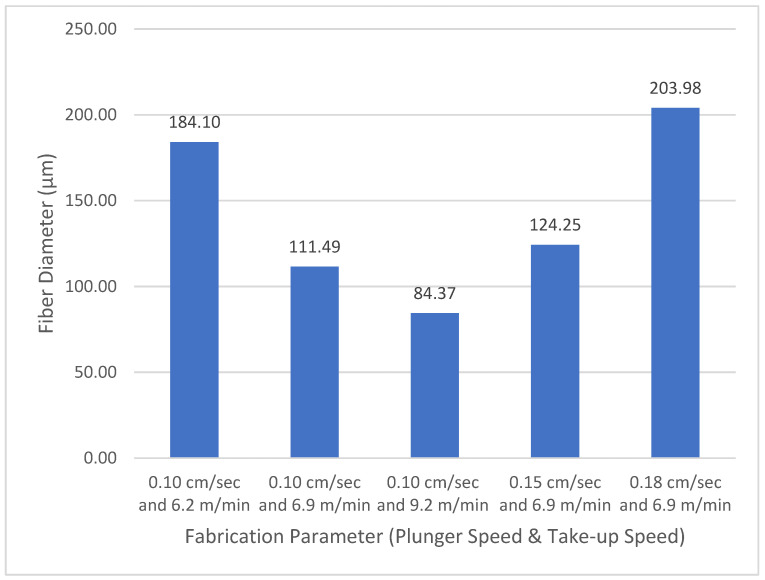
Correlation of plunger speed and take-up speed with polycarbonate fibre diameter.

Figure 4 shows the finest fibre diameter in this study was produced by setting the plunger speed at 0.10 m/min and the take-up speed at 9.2 cm/min, whereas the coarsest fibre diameter was produced by setting the plunger speed at 0.18 m/min and the take-up speed at 6.9 cm/min. This occurred because a high take-up speed leads to a rapid decrease in the fibre diameter [26]. 

### 3.3. Fibre Tenacity

**Figure 5:**
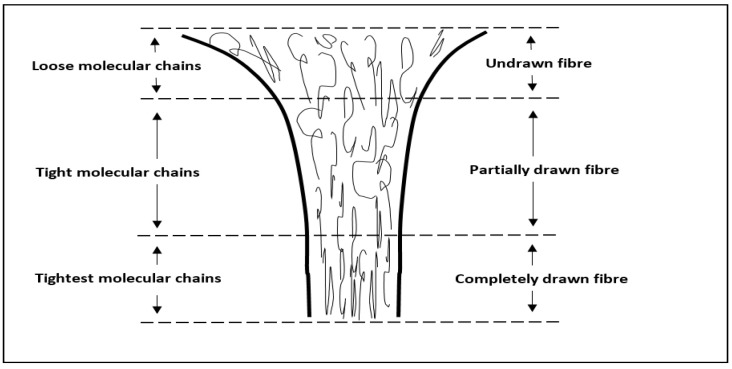
Change of molecular orientation in the take-up process.

**Figure 6:**
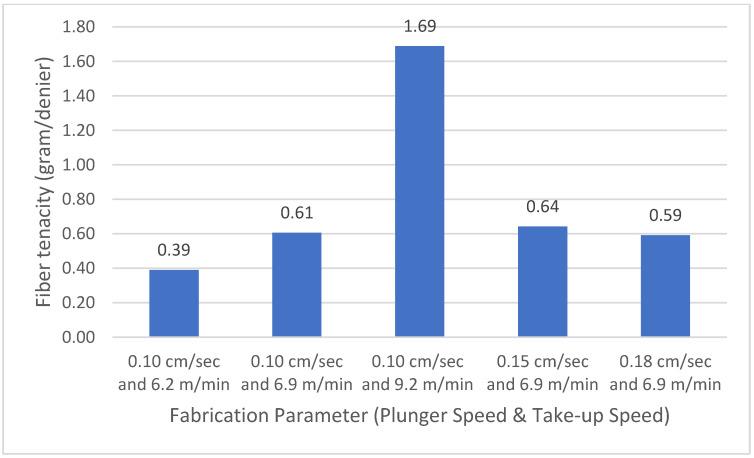
Correlation of plunger speed and take-up speed with polycarbonate fibre tenacity.

Fibre tenacity has an inversely proportional value to fibre diameter, a fine diameter of fibre leads to higher fibre tenacity, since an increase in the strain rate creates a better molecular chain orientation and alignment. An increase in molecular chain alignment leads to an increase in tenacity because the molecular chains become tighter, as shown in Figure 5 [27,28]. Figure 6 shows that the tenacity of fibre produced from the combination of the highest take-up speed and lowest plunger speed was 1.69 g/denier. The combination of the highest take-up speed and lowest plunger speed creates the highest strain rate, which leads to improvement of polymer molecular orientation degree. The improvement of polymer molecular orientation degree results in the increase in fibre tenacity.

### 3.4. Fabric Areal Density

**Figure 7:**
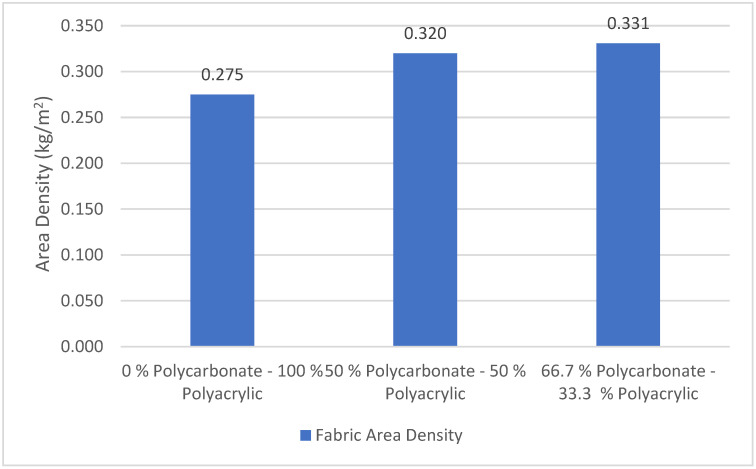
The areal density of polycarbonate–polyacrylic fabrics.

Fabric areal density is a measurement of mass per unit area of the fabric, it is affected by course per inch, wale per inch, yarn count, material insertion, and other knit structures that influence physical properties, such as shrinkage, water absorbency, and air permeability [29]. As can be seen in Figure 7, the 66.7% polycarbonate–33.3% polyacrylic fabric has the highest areal density, meanwhile, the 0% polycarbonate–100% polyacrylic fabric has the lowest areal density. This occurs because the 66.7% polycarbonate–33.3% polyacrylic fabric has two strands of recycled polycarbonate fibre insertion in every course, and this will improve fabric mass. Improvement of fabric mass affects the fabric’s physical properties, which is very important, especially for protective garments [30]. 

We determined that the 0% polycarbonate–100% polyacrylic fabric has the lowest areal density, more than 20% lighter than the areal density of the 66.7% polycarbonate–33.3% polyacrylic fabric. This may lead to poor cover opacity and bursting strength [31]. For some applications, lightweight knitted fabric such as the 0% polycarbonate–100% polyacrylic is preferred over other types of clothing [32]. 

### 3.5. Bursting Strength

**Figure 8:**
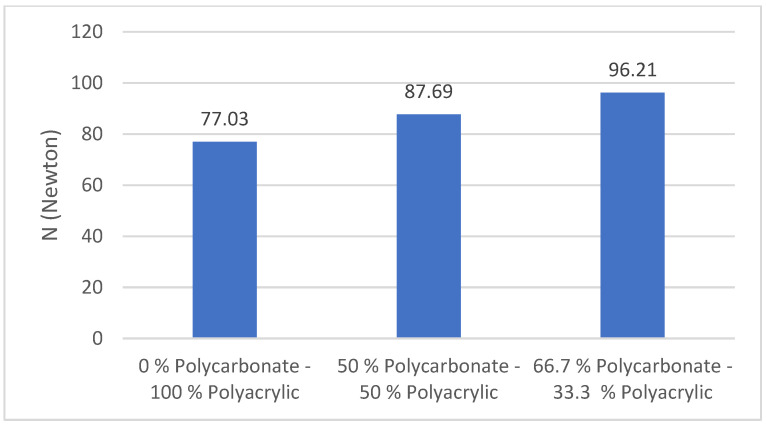
Bursting strength of polycarbonate–polyacrylic fabrics.

There are several important mechanical properties in fabric, one of them is bursting strength, which is generally associated with knitted fabrics, or breaking strength with woven fabrics [33,34]. Figure 8 shows that the 66.7% polycarbonate–33.3% polyacrylic fabric has a higher bursting strength compared with the 0% polycarbonate–100% polyacrylic fabric and the 50% polycarbonate–50% polyacrylic fabric. The bursting strength of the 66.7% polycarbonate–33.3% polyacrylic fabric was 25% higher than the 0% polycarbonate–100% polyacrylic fabric. This occurred because the 66.7% polycarbonate–33.3% polyacrylic fabric has recycled polycarbonate fibre insertion that absorbs external force during the bursting strength test. A high tightness factor and high areal density of the fabric structure leads to a high bursting strength [35]. According to this study, we found that the insertion of recycled polycarbonate fibres can improve the mechanical properties of a knit-fabric and that this is suitable for a high-performance textile application. 

### 3.6. Thermal Signature Reduction

A thermal signature is created through an apparent temperature differential between an object and its background [6]. Thermal signature is one of the most critical aspects to prevent or delay detection in low-light military camouflage operations. Military camouflage has at least two requirements that need to be managed; the near-infrared and the visible regions of the spectrum [36]. Temperature regulation is required for reducing the emitted infrared waves in military camouflage, lower emitted infrared waves lead to higher thermal insulation properties [37]. Therefore, it is important to select a material that has a low infrared emittance and high thermal insulation to reduce the thermal signature of camouflage textile. 

**Figure 9:**
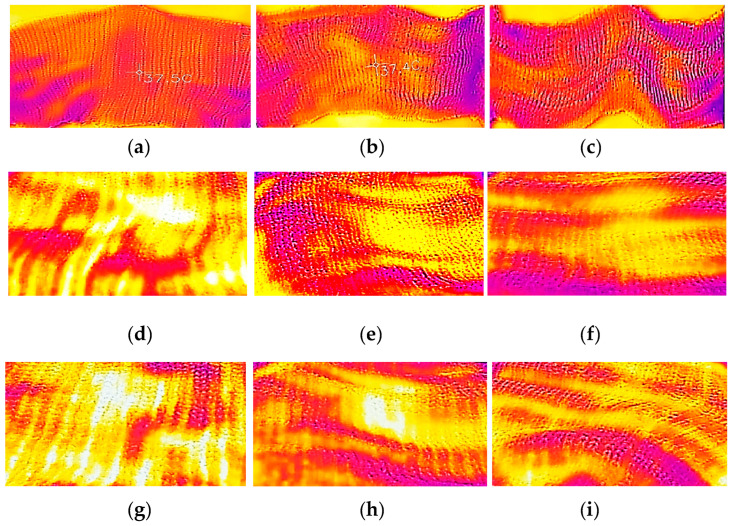
Thermography of knitted fabric: (**a**) 0% Polycarbonate–100% Polyacrylic on 40 °C; (**b**) 50% Polycarbonate–50% Polyacrylic on 40 °C; (**c**) 66.7% Polycarbonate–33.3% Polyacrylic on 40 °C; (**d**) 0% Polycarbonate–100% Polyacrylic on 68 °C; (**e**) 50% Polycarbonate–50% Polyacrylic on 68 °C; (**f**) 66.7% Polycarbonate–33.3% Polyacrylic on 68 °C; (**g**) 0% Polycarbonate–100% Polyacrylic on 100 °C; (**h**) 50% Polycarbonate–50% Polyacrylic on 100 °C; (**i**) 66.7% Polycarbonate–33.3% Polyacrylic on 100 °C.

Figure 9a–c show the thermal signatures of knitted fabrics for three different recycled polycarbonate fibre contents using a 40 °C heat source. Figure 9c, showing the fabric with the highest recycled polycarbonate content (67.7%), has a darker thermal signature compared with Figure 9b,c, which have lower recycled polycarbonate contents. The result was similar when the temperature of the heat source was increased, Figure 9d–f are the thermal signatures of knitted fabrics with various recycled polycarbonate fibre contents using a 68 °C heat source. The thermal signature of the knitted fabric in Figure 9d, which had the lowest recycled polycarbonate fibre content (0%), is brighter than that of the other knitted fabrics, which had a higher recycled polycarbonate content. The thermal signatures of the knitted fabrics on a 100 °C heat source are shown in Figure 9g–i. The knitted fabrics which had a higher recycled polycarbonate content produced darker thermal signatures, as shown by Figure 9i,j, which had 67% and 50% recycled polycarbonate content. Figure 9a–g have no recycled polycarbonate fibre insertion and all show brighter thermal signatures compared with the other fabric variations, especially Figure 9c,f,i, which had the highest amount of polycarbonate fibre insertion. It was found that applying recycled polycarbonate fibres as an insertion in polyacrylic knitted fabrics reduced the emitted infrared waves captured by a thermal imaging camera.

**Figure 10:**
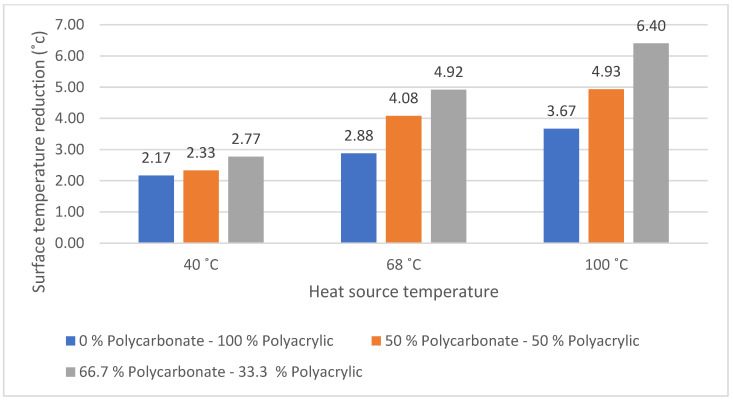
Surface temperature reduction in various polycarbonate-content fabrics.

Thermal signature reduction in infrared camouflage is reached by managing the surface temperature and the surface emittance of the object [38]. Decreasing the surface temperature of the object represents a reduction in the surface emittance and thermal signature. Figure 10 shows that the 66.7% polycarbonate–33.3% polyacrylic fabric has the highest surface temperature reduction in all heat source temperatures. At high operational temperature (100 °C), the surface temperature reduction in the 66.7% polycarbonate–33.3% polyacrylic fabric is almost 75% higher than the 0% polycarbonate–100% polyacrylic fabric. The effect is quite similar at other operational temperatures, for 40 °C and 68 °C heat source temperatures, the surface temperature reduction in the 66.7% polycarbonate–33.3% polyacrylic fabric is 27.6%, and 70.1% higher than the 0% polycarbonate–100% polyacrylic fabric. This is caused by the use of recycled polycarbonate fibre as an insertion in polyacrylic knitted fabric, since polycarbonate has a low thermal conductivity of 200 mW/m*K compared with polyacrylic yarn which has 310 mW/m*K of thermal conductivity [39,40]. The most substantial differences can be observed using a heat source temperature of 100 °C, where the knitted fabric thermal insulation is increased to 74.5% in the 66.7% polycarbonate–33.3% polyacrylic fabric. A higher operational temperature will lead to a higher thermal insulation difference. The thermal insulation of the fabric is proportional to operational temperature because thermal conductivity is dependent on temperature [39].

The surface temperature reduction is directly proportional to the recycled polycarbonate fibre content, higher recycled polycarbonate fibre content leads to the increases in surface temperature reduction. This result confirms the advantage of applying recycled polycarbonate fibre to reduce the surface temperature and thermal signature of the fabric. 

## 4. Conclusions 

This research produced and evaluated recycled polycarbonate fibre from multiwall polycarbonate roofing-sheet waste. It was found that multiwall polycarbonate roofing-sheet waste was a potential material for synthetic fibres due to its spinnability. High take-up speeds produced fine fibres, whereas high plunger speeds lead to lower strain rates and created coarse fibrea. The coarsest fibre diameter in this study was produced by setting the plunger speed at 0.18 m/min and the take-up speed at 6.9 cm/min, whereas the finest fibre diameter was produced by setting the plunger speed at 0.10 m/min and the take-up speed in 9.2 cm/min; this setting also produced the highest fibre tenacity of 1.69 g/denier. The take-up speed value was proportionally linear to the resulting tenacity of fibre. 

The insertion of recycled polycarbonate fibre can improve the mechanical properties of knitted fabrics and it is suitable for high-performance textile applications, the bursting strength of the 66.7% polycarbonate–33.3% polyacrylic fabric was 25% higher than the 0% polycarbonate–100% polyacrylic fabric. The fabric which had a higher recycled polycarbonate fibre content had a higher surface temperature and thermal signature reduction at all temperatures. The surface temperature reduction in the 66.7% polycarbonate–33.3% polyacrylic fabric was almost 75% higher than the 0% polycarbonate–100% polyacrylic fabric. We found that applying recycled polycarbonate fibres as a fibre insertion in polyacrylic knitted fabrics reduced the emitted infrared waves captured by a thermal imaging camera.
